# Prenatal Environmental Metal Exposure and Preterm Birth: A Scoping Review

**DOI:** 10.3390/ijerph18020573

**Published:** 2021-01-12

**Authors:** Rasheda Khanam, Ishaan Kumar, Opeyemi Oladapo-Shittu, Claire Twose, ASMD Ashraful Islam, Shyam S. Biswal, Rubhana Raqib, Abdullah H. Baqui

**Affiliations:** 1 International Center for Maternal and Newborn Health, Department of International Health, Johns Hopkins Bloomberg School of Public Health, Baltimore, MD 21205, USA; rkhanam1@jhu.edu (R.K.); ooladap2@alumni.jh.edu (O.O.-S.); 2 Department of Chemistry, Georgetown University, Washington, DC 20057, USA; ik376@georgetown.edu; 3 Welch Medical Library, Johns Hopkins University School of Medicine, Baltimore, MD 21205, USA; ctwose@jhmi.edu; 4 Projahnmo Research Foundation, Dhaka 1213, Bangladesh; aislam@prfbd.org; 5 Department of Environmental Health and Engineering, Johns Hopkins Bloomberg School of Public Health, Baltimore, MD 21205, USA; sbiswal@jhu.edu; 6 International Center for Diarrheal Disease Research, Mohakhali, Dhaka 1212, Bangladesh; rubhana@icddrb.org

**Keywords:** lead, mercury, cadmium, arsenic, prenatal exposure, preterm birth, scoping review

## Abstract

Preterm birth (PTB) and its complications are the leading causes of under-five year old child deaths, accounting worldwide for an estimated one million deaths annually. The etiology of PTB is complex and multifactorial. Exposures to environmental metals or metalloids are pervasive and prenatal exposures to them are considered important in the etiology of PTB. We conducted a scoping review to determine the extent of prenatal exposures to four metals/metalloids (lead, mercury, cadmium and arsenic) and their association with PTB. We reviewed original research studies published in PubMed, Embase, the Cochrane Library, Scopus, POPLINE and the WHO regional indexes from 2000 to 2019; 36 articles were retained for full text review. We documented a higher incidence of PTB with lead and cadmium exposures. The findings for mercury and arsenic exposures were inconclusive. Metal-induced oxidative stress in the placenta, epigenetic modification, inflammation, and endocrine disruptions are the most common pathways through which heavy metals and metalloids affect placental functions leading to PTB. Most of the studies were from the high-income countries, reflecting the need for additional data from low-middle-income countries, where PTB rates are higher and prenatal exposure to metals are likely to be just as high, if not higher.

## 1. Introduction

Preterm birth (PTB), defined as birth before 37 completed weeks of gestation, is a major global public health problem due to its high incidence and associated high morbidity, mortality, and long-term disability [1,2]. Each year, about 15 million babies or 11.1% of all live birth babies are born preterm worldwide [3]. The incidence of PTB ranges from about 5% in many European countries to up to 18% in some South Asian and sub-Saharan African countries with the highest burden (60%) in South Asia and sub-Saharan Africa [3]. High-income countries may also experience a high burden; the United States is one of the top ten countries in the world with the greatest number of PTB [4,5]. The mortality risk is much higher among preterm babies compared to term babies. Globally, PTB and its complications are the leading cause of death among children under 5 years of age, responsible for approximately 1 million deaths in 2015 [3,6].

The causes of PTB are multifactorial and most cases occur due to a complex interplay of genetic, environmental, and host factors. A plethora of socio-economic, nutritional, medical, obstetric, and environmental factors have been shown to increase the risk of PTB. However, the precise etiology of PTB remains poorly understood [5,7].

Exposure to heavy metals during pregnancy is one concern. Many studies over the last few decades have examined the association of exposures to environmental heavy metals, as well as other environmental chemicals contaminants, with adverse pregnancy outcomes (APOs), including miscarriage, stillbirth, PTB, small for gestational age and low birth weight. Several earlier reviews also highlighted the association of exposures to environmental pollutants including heavy metals with adverse pregnancy outcomes [8,9,10,11,12,13,14,15]. The findings of the studies that examined the association between exposure to environmental heavy metals and metalloids during pregnancy and PTB are mixed [5].

We have conducted a focused scoping review to examine the association of prenatal exposure of selected environmental heavy metals and metalloids with the incidence of PTB. We also examined the potential pathways of environmental metal exposure related PTB. The objective of the review is to better delineate the burden of exposure to lead (Pb), mercury (Hg), cadmium (Cd) and arsenic (As), their association with PTB, and possible mechanisms to inform future research and prevention strategies.

## 2. Materials and Methods

### 2.1. Data Sources and Search Criteria

Search strategies were constructed to identify peer reviewed, published literature addressing the association between environmental exposures to selected heavy metals and arsenic, with human PTB. Specifically, the searches were constructed to identify studies of contamination by Pb, Hg, Cd and As. Databases selected for inclusion were PubMed, Embase, the Cochrane Library, Scopus, POPLINE and the WHO regional indexes. Since environmental heavy metal contaminations exist in countries regardless of their level of economic and political development, no geographic limits were used. Searches were run in May through August 2019. The search was limited to the year 2000 forward.

The initial search strategy was developed iteratively for the PubMed database by an information professional, Claire Twose (CT) with input from the lead authors. Once all authors were satisfied with both the breadth and specificity of the results, this strategy was translated for the other five databases by CT.

The final PubMed strategy, run on the legacy PubMed interface, was:

(“metals, heavy”[Mesh] OR heavy metals[tw] OR cadmium[tw] OR chromium[tw] OR copper[tw] OR lead[tw] OR manganese[tw] OR mercury[tw] OR zinc[tw] OR “arsenic”[Mesh] OR arsenic[tw])

AND

(“premature birth”[Mesh] OR premature birth*[tw] OR preterm birth*[tw] OR pre-term birth*[tw] OR infant, premature[mesh] OR obstetric labor, premature[mesh] OR premature labor[tiab] OR premature labour[tiab] OR preterm labor[tiab] OR pre-term labor[tiab] OR preterm labour[tiab] OR pre-term labour[tiab])

Data range: Publication date from 1 January 2000

Search results were downloaded into EndNote to facilitate removal of duplicate citations and the resulting unique set of citations was uploaded to the online tool Covidence for title and abstract as well as full text screening.

### 2.2. Inclusion and Exclusion Criteria

The inclusion criteria for title and abstract screening were: Full-text articles written in English, human studies, original studies that described exposure to lead, mercury, cadmium, and arsenic, to women during pregnancy and examined the association with preterm/premature birth or adverse pregnancy outcomes. The studies were excluded if not written in English, non-human studies, and if there was no mention of arsenic, cadmium, lead, or mercury and preterm birth/premature birth/adverse pregnancy outcomes in the study titles or abstracts.

### 2.3. Screening of Papers

Three reviewers (RK, IK, OOS) participated in the review process. All titles and abstracts were reviewed independently by two of the three reviewers with conflicts resolved by the senior author (AHB). Before starting the screening process, a calibration exercise was performed to ensure standardized review of the papers by the reviewers. For standardization, two of the three reviewers screened each paper independently from the PubMed searches and compared until >90% agreement was achieved. Similar exercise was performed before the full text data abstraction. During calibration, the disagreements were discussed between the reviewers to resolve.

### 2.4. Data Abstraction

The papers retained for full review were distributed among the three reviewers in a way that two reviewers independently reviewed each full-text article and extracted relevant data. The same inclusion and exclusion criteria were used for the full text screening. Data were abstracted from original studies only. Review papers were separately reviewed to find out if our search failed to capture any original study that was captured by an earlier review. The following data were extracted: country where the study was conducted, study participants, study design, specimen type and timing of collection, main findings, and controls variables. Information on possible mechanisms of PTB due to exposures to metals was separately extracted and presented in the discussion section of the manuscript.

### 2.5. Data Charting Process

The data abstraction for this scoping review was conducted separately for four metals/metalloids: Pb, Hg, Cd and As. Data abstraction was performed using custom designed data extraction table using google doc. Data were later auto transferred into an excel sheet. The authors then created tables followed by a narrative synthesis of the results separately for each metal.

### 2.6. Quality Assessment

In addition, we assessed the methodological quality of the papers using the Newcastle-Ottawa Scale (NOS) for Quality Assessment [1,6,7,8,9,10,11,12,13,14,15,16,17,18,19]. Two of the three reviewers independently assessed each paper. The NOS guideline use three broad predefined criteria, some of which require additional specification for a specific review: (1) the selection of the study population (0–4 stars possible); (2) the comparability of the study groups (0–2 stars possible); and (3) the determination of exposure for case-control and outcome of interest for cohort or cross-sectional studies (0–3 stars possible). The total score for each study ranges from 0 to 9. Disagreements between the two reviewers were resolved through discussion. Based on the total scores, studies were categorized into high quality (7 to 9), moderate quality (4 to 6) and low quality (1 to 3). The quality of ecological studies was not assessed.

## 3. Results

**Figure 1:**
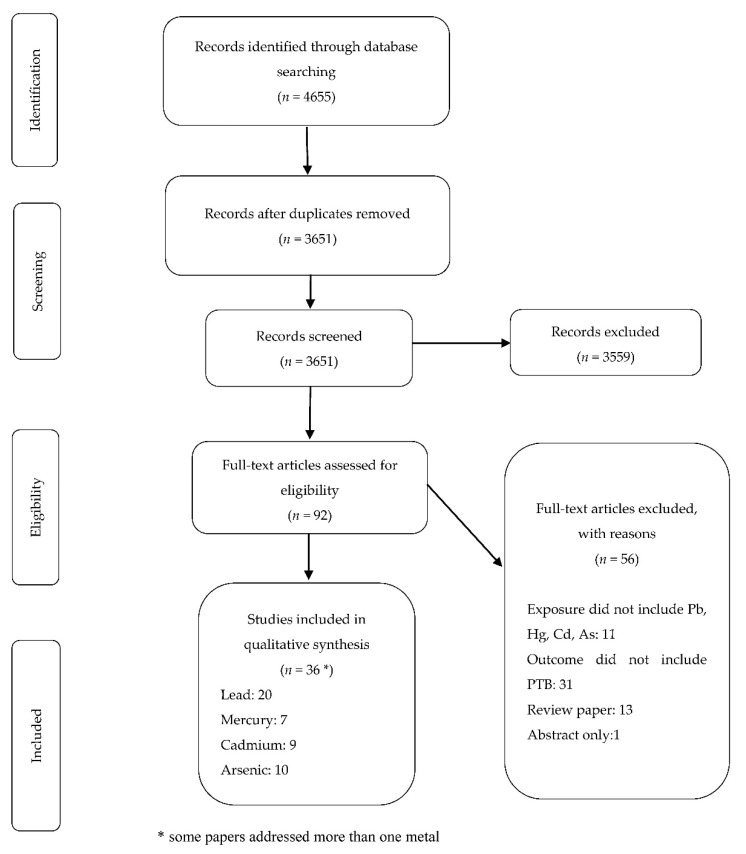
PRISMA flow diagram of selection of papers and data extraction process.

The database searches retrieved a total of 4655 citations. As shown in the PRISMA flow diagram [20], 3651 unique titles and abstracts were retained for screening after removing the duplicates. After screening the titles and abstracts, 92 papers were selected for full text screening, with 36 ultimately included in the review (Figure 1).

Of the papers excluded at the full text screening stage, 31 did not report results on PTB, 13 were review papers, 11 did not address the metals of interest in this review and one did not have sufficient published information to be analyzed. The findings of association of each of the four selected metals/metalloid with PTB and the quality of the papers are presented separately.

### 3.1. Lead and PTB

We identified 20 papers that assessed the relationship between exposure to environmental metal Pb and PTB (Table 1). The study sizes varied substantially, ranging from 50 to 169,878 mother-infant pairs. Several studies presented Odds Ratios along with 95% confidence intervals while others presented the median or mean lead level in term birth vs. PTB. Regarding potential confounders, 13 papers [21,22,23,24,25,26,27,28,29,30,31,32,33] adjusted for one or more potential confounders, while seven papers [34,35,36,37,38,39,40] did not. Sixteen out of 20 studies were of high quality, while the remaining studies were of moderate quality (Table 1).

Three studies from China reported significantly increased risk of PTB associated with prenatal exposure to Pb. Cheng et al. studied 7299 pregnant women, categorized them into terciles based on creatinine-adjusted urinary lead levels measured during pregnancy: Low (≤2.29 g/g Cr), Medium (2.29–4.06 g/g Cr), and High (>4.06 g/g Cr). Compared to women with the lowest tercile Pb level, the risk of PTB was about 40% higher among medium-tercile women (AOR, 95% CI: 1.43, 1.07 to 1.89) and about two-fold higher among women in the highest tercile (AOR, 95% CI: 1.96, 1.31 to 2.44) [23]. Another study of 3125 pregnant women reported mean serum Pb level of 1.50 µg/dL with a range from 0.020 to 5.46 µg/dL. Using serum Pb level, the women were classified into three groups: Low-Pb (L-Pb, <1.18 µg/dL), Medium-Pb (M-Pb, 1.18–1.70 µg/dL), and High-Pb (H-Pb, ≥1.71 µg/dL) [26]. Compared to women with low-Pb level, the risk of PTB was more than two fold higher in women with medium-Pb (AOR, 95% CI: 2.33, 1.49 to 3.65; *p* < 0.001) and about 3-fold higher among women with H-Pb (3.09; 2.01, 4.76; *p* < 0.001) [26]. A third study reported similar association [32]. All three studies demonstrated a dose-response relationship between Pb exposure and PTB [23,26,32].

Five studies conducted in the USA reported variable association between higher levels of Pb and PTB. Jelliffe-Pawlowski et al. examining deliveries between 1996–2002 in a population of primarily Hispanic women from California, observed that the blood Pb levels ≥10 μg/dL during pregnancy was associated with significantly higher risk of PTB (*n* = 262, OR, 95% CI: 3.2, 1.2 to 7.4) [25]. Rabito and colleagues reported that a 0.1 unit increase in maternal blood lead in the second trimester was associated with pre-term birth (OR, 95% CI:1.66, 1.23 to 2.23, *p* < 0.01). Similarly, third trimester maternal blood lead was also associated with PTB (OR, 95% CI: 1.24, 1.01 to 1.52, *p* = 0.04) [39]. On the other hand, in a large study (*n* = 43,288) in USA, Zhu et al. found that in the highest quartile of Pb exposure (3.1–9.9 μg/dL), there was no significant increase in odds of PTB compared to the lowest quartile (≤1 μg/dL) [33]. The average Pb exposure was 2.1 μg/dL, which was low compared to other studies and the blood samples were obtained throughout the pregnancy [33]. The two other studies from USA did not find any association between prenatal exposure to Pb and PTB [22,27].

Taylor and colleagues found in a study conducted in the UK that blood Pb level ≥ 5 µg/dL significantly increased the risk of PTB (AOR, 95% CI: 2.00, 1.35 to 3.00) [28]. Cantonwine et al., in a study in Mexico, examined the relationship between trimester specific association of Pb exposure during pregnancy and risks of PTB. The study observed that high blood Pb levels during both the first and second trimesters could predict PTB, however the strongest association was observed with 2nd trimester blood Pb (OR, 95% CI: 1.75, 1.02 to 3.02) [21].

A study in Tehran (Iran) examined maternal blood Pb levels collected during first trimester of pregnancy and reported a significantly higher Pb level in mothers who delivered preterm babies compared to women who delivered at term (OR, 95% CI: 1.41, 1.08 to 1.84, *p* < 0.05) [30]. The average blood Pb level was 3.5 mg/dL, which suggest that blood Pb even at ‘acceptable’ levels (≤10 µg/dL), could be a risk factor for PTB [30]. Several studies observed an association between Pb level in cord blood with PTB [24,35]. However, the results of studies that examined association between cord blood Pb level and PTB were conflicting [24,37,40].

Similarly, findings of the studies that examined association of placental Pb levels with PTB were conflicting. Ahamed et al. in a study in India examined Pb concentrations in placental tissue and its outcome with PTB. Placental lead level was significantly higher in women with preterm deliveries compared with the full-term (0.39 g/g vs. 0.27 g/g, *p* < 0.05) [34]. Another study in Spain by Falcon et al. found significantly higher Pb levels in placenta of those with premature rupture of the membranes and preterm delivery, compared to normal pregnancies (*n* = 89) [36]. The other study conducted by Irwinda et al. in Indonesia found significantly higher Pb level in the placental tissue among preterm than term group (0.81(1.43) ng/g vs. 0.02(0.01) ng/g, *p* value = 0.009) [37]. Although the study showed low levels of Pb in maternal serum (*p* = 0.177) and cord blood (*p* = 0.244) in the PTB group than from the term group. However, this was not statistically significant [37]. The other study by Freire, C., et al. found no significant association between placental Pb levels and PTB. The Pb was detected only in 13% of the placental samples, which might explain the lack of an association with PTB [24]. Three other studies observed no association between placental Pb level and PTB [29,31,40].

### 3.2. Mercury and PTB

We identified seven papers that assessed the relationship between exposure to environmental mercury and PTB [24,29,37,40,41,42,43] (Table 2). Five of the seven studies had a small sample size. Four studies adjusted for possible confounders. Six studies were assessed to be of high quality and six studies were from USA or Europe. The findings of the studies were inconsistent.

A large study from Japan did not observe any association with maternal blood Hg level and PTB [29]. However, Hg concentrations in this study were lower than the reported levels in Japanese population [44]. Burch et al. conducted a large study in all live births during 1995 to 2005 in South Carolina (USA) investigating association of Hg level in fish of different population with PTB. The areas were categorized into four quartiles depending on total mercury concentrations in the fish. PTB rates were 10–18% higher among African American women living in areas with the higher total fish mercury concentrations compared to the area with lowest concentration [43]. This was an ecological study subject to confounding.

A small study conducted in 50 African American mother-infant pairs included in the Boston birth cohort study, USA observed higher Hg level in maternal serum, maternal RBC, cord plasma and cord RBC of preterm babies compared to term babies [41]. The mean (95% CI) of Hg concentration in mother’s RBCs (µg/L) who delivered a preterm baby [1.86 (1.49–2.33)] was higher compared to mothers who delivered a term baby [1.37 (1.21–1.55)]. Similarly, Hg level in cord RBC’s (µg/L) was higher for PTB [2.22 (1.67–2.96)] than from term births [1.65 (1.46–1.86)] [41]. However, this study did not adjust for any potential confounders. The study documented higher mean blood Hg level than the National Health and Nutrition Examination Survey (NHANES) 2001–2002 estimate (0.83 µg/L in whole blood). The Hg concentration in cord blood RBCs was 1.5 times higher than in the mother’s RBCs, indicating a high degree of maternal–fetal transfer of Hg [41].

Another study from Jakarta (Indonesia), measured mercury concentrations in maternal serum, placenta, and umbilical cord obtained at birth [37]. The study found significantly higher mercury concentrations in placentas (ng/g) of women who had a preterm delivery [20.47 (41.35)] compared to those who had term delivery [0.20 (0.17), *p* = 0.019]. Nevertheless, no significant difference in Hg concentration was found for the maternal serum (*p* = 0.178) and cord blood sample (*p* = 0.461) [37].

The remaining studies we reviewed for this paper showed no association between exposure to mercury and preterm delivery [24,42]. Bashore et al. examined the association between maternal urinary Hg and cord blood Hg levels and PTB, and found no association between maternal exposure to Hg and PTB [42]. In Spain, Freire et al. conducted a study in 327 mother-infant pairs from 2000 to 2008. Mercury concentration was measured in placental specimens. The study reported median (IQR) concentration of Hg was 4.427 (0.016–17.11) ng/g but did not find any association between the detection level of Hg [1.36 (CI: 0.66–6.42)] with PTB [24].

### 3.3. Cadmium and PTB

We identified nine papers that assessed the relationship between exposure to environmental cadmium and PTB [24,29,31,38,40,45,46,47,48] (Table 3). All but one of the studies had a reasonable sample size. Two studies did not adjust for possible confounders [38,40]. Four studies were from the USA [46] or Europe [24,38,40], three were from China [45,47,48] and one was from each of Japan [29] and Myanmar [31]. The studies used varied specimen type including maternal blood, urine and meconium.

Two of the three studies from China measured Cd in maternal urine samples and one measured it in a maternal blood sample. All three studies observed a high level of Cd and demonstrated significant association of Cd exposure with PTB [45,47,48]. Huang et al. in China measured urine Cd levels and reported increased risk of preterm low birth weight (PLBW) among women with a Cd level of ≥0.70 μg/g [45]. Another study by Wang et al. measured Cd in maternal blood and observed that PTB was about three times higher (mean, range 2.02, 4.50; *p* < 0.001, *n* = 3254) among women with high Cd levels (≥0.95 mg/L) compared to women with a low level (<0.65 mg/L) [47]. The third study also observed an association between urine Cd level and PTB (AOR, 95% CI: 1.78, 1.45 to 2.19) [48].

A study from Turkey by Ozsoy et al. measured meconium Cd levels and found a significant association (*p* < 0.001) between meconium Cd level and PTB [38]. A large study from Japan observed similar association between maternal blood Cd measured during 14–39 weeks of pregnancy and PTB [29]. In contrast, Freire et al. observed a negative association between placental Cd level and PTB. A 10% increase in Cd levels was associated with a 9% lower risk (95% CI: 0.84 to 0.99, *p* < 0.05) of PTB [24]. Three remaining studies did not observe a significant association between exposure to Cd during pregnancy and PTB [31,40,46].

### 3.4. Arsenic and PTB

We identified 10 peer-reviewed papers that investigated the association of arsenic exposure with PTB [24,31,49,50,51,52,53,54,55,56] (Table 4). A majority were conducted in Asian developing countries: three papers were from Bangladesh, two from China, and one from each of Taiwan and Myanmar. Papers from western countries included two from the United States and one from Spain. A majority of the studies assessed ground water in the form of tube wells or dug wells while one determined arsenic level from safe drinking water [49,50,51,52,53,54,56]. The remaining studies measured arsenic levels in maternal serum [55], placental tissue [24], and maternal urine [31].

Ahmad et al., in a study in Bangladesh, found the PTB rate to be significantly higher among women living in areas with high exposure to As (≥0.10 mg/L in well water) compared to women residing in low exposure areas (<0.02 mg/L in well water) (*n* = 192) [49]. Another study in Bangladesh by Rahman et al. measured As concentration in tube well water and in maternal toenails [53]. The results indicated that every unit increase in the natural log of arsenic exposure was associated with a 12% (RR, 95% CI: 1.12, 1.07 to 1.17; *p* < 0.001) higher risk of PTB after adjusting for common confounders. The risk ratio for toenail arsenic exposure among PTB was 1.13 (RR, 95% CI: 1.13, 1.03 to 1.24) [53]. The third study in Bangladesh found a 84% higher risk of PTB among women who drank tube well water containing more arsenic per liter than Bangladesh’s safe drinking water standard (50 µg As/L); however this finding was not statistically significant (OR, 95% CI: 1.84, 0.81 to 4.17) [51].

Wang et al. in a study in China among 3194 pregnant women reported higher risk of moderate-to-late PTB of 1.47 (95% CI: 1.03 to 2.09; *p* = 0.034, *n* = 3194) in high-As group as compared with low-As group [55]. In a study based in the USA, Almberg and colleagues reported an 8−10% increase in the odds of PTB per 1 μg/L increase in arsenic in drinking water for counties with <10% and <20% private well use [50]. Several other studies that observed low arsenic levels in their populations did not observe any significant association with PTB [24,31,52].

## 4. Discussion

This scoping review examined the association of prenatal exposures to Pb, Hg, Cd and As with the incidence of PTB and demonstrated a higher incidence of PTB with increased levels of Pb and Cd exposures. The findings for Hg and As exposures were inconclusive.

The association between prenatal Pb exposure and adverse pregnancy outcomes, including PTB has been well studied. Several prior reviews suggested a positive association [8,9,10,11,12]. Despite some of the inconsistent findings in the 20 papers that we identified; a majority of the studies documented that high prenatal maternal Pb level during pregnancy was associated with increased risk of PTB. Studies that did not show an association were either small or conducted in populations with a generally low Pb level or had one or more methodological concerns e.g., did not adjust for confounding variables. Some studies suggested that there is no threshold for Pb above which Pb exposure leads to PTB. Maternal blood lead even at ‘acceptable’ levels, could be a risk factor for PTB [30,39]. The Centers for Disease Control (CDC) also recently published a statement indicating that there is no threshold below which lead exposure is acceptable and has specifically called for additional research into adverse pregnancy outcomes related to prenatal exposure [57,58].

Most of the lead studies were from high income countries. There is a paucity of data from low- middle-income countries (LMICs) of South Asia and sub-Saharan Africa where the PTB birth rates are high and risk of exposure to metals during pregnancy, including Pb is also likely to be high. The metal Pb was detected from many different types of specimens including maternal blood, maternal serum, maternal urine, cord blood and placental tissue. The detection level of Pb from urine, a noninvasive sample, was more than 99% in two studies included in this review [23,32]. The use of urine sample will make studies more feasible in LMIC settings. We conclude that in populations with generally high environmental Pb level, Pb exposure is a risk factor for PTB. Additional research, particularly in LMICs to quantify the burden and to develop strategies to reduce the exposure is needed.

As alluded to earlier, our review was inconclusive about the association between prenatal Hg exposure and PTB. Five of the seven studies included in this review had small sample sizes. There was only one study from an LMIC—Indonesia—that found a significantly higher Hg concentration in placenta of women who had a preterm delivery compared to those who had term delivery, but a similar association was not observed between Hg level in maternal serum or cord blood sample and PTB [37]. Studies are needed, particularly in LMICs, to investigate the extent of Hg exposure and its association with PTB. Studies should also investigate the optimal specimen type that should be used to measure exposure to Hg.

The majority of the studies included in this review observed a positive association between maternal Cd exposure and PTB, a few did not, and one study observed a negative association [24]. It is possible that the association between prenatal Cd exposure and PTB is context specific; only populations with high environmental Cd exposures experience this problem. All three studies from China observed high Cd level and association of Cd exposures with PTB [45,47,48].

Our review was also inconclusive about the association between As exposure and PTB. There was considerable evidence to support the association between chronic exposure to moderate-to-high levels of As (>50 ppb) and APOs including spontaneous abortion/miscarriage, stillbirth, and low birth weight. Data on PTB is sparse with inconsistent findings [10,49,59]. Moreover, a majority of the studies were ecological; this study design is subject to confounding.

The pathways or mechanisms through which heavy metals and metalloids affect placental functions and pregnancy outcomes are not adequately understood. In animal and limited human studies, some metals have been shown to have the ability to cross the placenta causing reproductive toxicities and APOs [60,61,62,63]. Currently available data suggest that some common pathways are involved: (1) Oxidative stress caused by an imbalance between the production of reactive oxygen species (ROS), reactive nitrogen species (RNS) (e.g., superoxide, nitric oxide) and the ability of the antioxidant defense systems (e.g., Superoxide Dismutase, glutathione, catalase) to neutralize them. The heavy metals act as catalysts in the redox reactions resulting in uncontrolled production of ROS and RNS, which in turn can lead to lipid peroxidation, DNA damage, and cell cytotoxicity [12,28,64,65]; (2) Epigenetic modification via DNA methylation, acetylation, ubiquitination and histone modifications, or disruption in micro RNA expression, all of which can lead to inflammatory responses [66,67]; (3) Inflammation may also occur due to increased activation of innate cells and T helper cells and release of pro-inflammatory cytokines/chemokines—Intrauterine inflammation could affect placental function, in particular trophoblast proliferation and differentiation, increasing vascular reactivity [65,68]; and (4) Endocrine disruption; many heavy metals are endocrine disruptors [69] which can interrupt signaling pathways for hormones, e.g., estrogen and progesterone [8,70,71]. Oxidative stress, inflammation and hormonal regulation in pregnancy are all closely linked to each other. The downstream consequences of such cascade of events either in the maternal systemic environment or the intrauterine milieu may initiate spontaneous preterm labor.

Preventive strategies that were successful in reducing the adverse consequences of exposures to metals include dietary supplements rich in vitamin and minerals. Diets play an important role in protecting against Cd and Pb toxicity. Fruits and vegetables such as tomatoes, berries, onion, garlic and grapes including iron, calcium, selenium, zinc, vitamins B, C, and E are natural antagonists to Cd and Pb toxicity [72] and should be consumed for both the prevention and alleviation of Cd and Pb toxicity. Experimental and human studies have demonstrated that deficiencies of certain micronutrients such as cyanocobalamin and folate disrupt normal epigenetic programming, giving rise to the possibilities of nutrition-based treatment of As-exposed population [73,74,75,76]. Arsenic metabolism includes methylation of inorganic arsenic (iAs) to mono- and dimethyl arsenical species (MMA, DMA) facilitating excretion of arsenic through urine. Arsenic methylation is influenced by nutrients such as folate and B12. Dietary folate intake and folate status have been positively associated with arsenic methylation capacity [74]. In randomized control trials, folic acid supplementation was shown to increase urinary excretion of DMA and reduced blood As levels [77,78,79] even though the results are not always consistent. Several studies showed that zinc and iron supplementation have positive impact to mitigate Pb toxicity [80,81,82,83]. However, data from the pregnancy period in relation to intervention/prevention are scarce [84,85,86,87].

Literature evaluating the synergistic or competitive effects of combined metal exposures on pregnancy and birth outcomes is limited. Accumulating evidence suggest that exposure to mixtures of toxic metals or other chemicals, may have additive, synergistic or antagonistic effects [88]. Exposure to high concentration of certain metals during pregnancy were associated with increased cardio-metabolic risk in childhood [89]. One study reported that combined prenatal metal exposures including lead, mercury and cadmium during pregnancy demonstrated a marked effect on neurodevelopment of 6 months old infants with significant interaction between lead and mercury [90]. Prenatal exposure to certain heavy metals was found to impart both synergistic and interactive effects of certain heavy metals on cognitive and motor impairment in 4–5 years old children [91].

## 5. Conclusions

Despite some inconsistencies in the findings across studies, there is overwhelming evidence that Pb and Cd exposures are associated with the incidence of PTB. The data regarding Hg and Arsenic are less certain requiring additional investigations. The discrepancies could have also resulted due to variation in the study design (e.g., cross-sectional, retrospective), types of samples (e.g., biological, environmental) and timing of collection of samples for measuring exposure (e.g., early, mid or late gestation).

Pb, Hg, Cd and As are naturally occurring elements, ubiquitous in the environment, and are well-known environmental pollutants due to their toxicity, persistence in the environment, and bio-accumulative nature. Humans are exposed to toxic metals through a variety of routes including ingestion, inhalation, and absorption through the skin. Numerous studies have reported elevated levels of multiple heavy metals in bio-specimens from individuals in LMICs compared to those from developed countries [92,93]. Additional studies are needed to examine the extent of the exposures to these metals and their association with PTB. Studies are also needed to further delineate the mechanisms through which metal exposures lead to PTB allowing design of preventive strategies, particularly in LMICs.

## Figures and Tables

**Table 1:** Association between Lead and PTB.

Reference	Country	Study Design	Sample Size	Specimen Type and Timing	Control Variables Adjusted	Association with PTB Odds Ratio or Relative Risk (95% CI), *p* Value	Qualitative Assessment Score
Cantonwine et al., 2010 [21]	Mexico	Cohort	*n* = 235	Maternal blood at 2nd trimester	Maternal age, education, prior adverse birth outcome, cigarette smoking during pregnancy, infant sex	Mean ± SD: 6.3 ± 4.3 μg/dL OR, 95% CI for one SD increase in Pb: 1.75, 1.02 to 3.02	8
Ahamed et al., 2009 [34]	India	Case-control	*n* = 60	Placental tissue	-	Term vs. Preterm Mean ± SD: 0.27 ± 0.15 μg/g vs. 0.39 ± 0.2 μg/g; *p* < 0.05	7
Berkowitz et al., 2006 [22]	USA	Ecological	*n* = 169,878	Pb level in air	Maternal age, infant’s sex, birth order, prior stillbirth	No significant association	-
Cheng et al.,2017 [23]	China	Cohort	*n* = 7299	Maternal urine before delivery	Maternal age, occupation, BMI, parity, passive smoking, pregnancy-induced hypertension, urinary concentration of cadmium and arsenic.	Pb concentration in Tercile2 (2.29–4.06 µg/g Cr): AOR, 95% CI: 1.43, 1.07 to 1.89 Tercile3 (>4.06 µg/g Cr): AOR, 95% CI: 1.96, 1.31 to 2.44, *p* < 0.01	8
El Sawi et al., 2013 [35]	Egypt	Cohort	*n* = 100	Cord blood	-	Mean ± SD 8.77 µg/dL ± 4.03. High Pb group (≥10 µg/dL^−1^) vs. Low Pb grp (<10g/dL^−1^), 33.3% vs. 0.4%, *p* < 0.001	6
Falcon et al., 2003 [36]	Spain	Cross-sectional	*n* = 89	Placental tissue	-	Term vs. PTB Mean ± SD Pb (ng/g) 103.2 ± 49.5 vs. 153.9± 71.7, *p* = 0.004	7
Freire et al., 2019 [24]	Spain	Cohort	*n* = 327	Placental tissue	Education, newborn sex, level of other metals (As, Cd, Mn, Cr)	No significant association	8
Irwinda et al., 2019 [37]	Indonesia	Cross-sectional	*n* = 51	Maternal blood, placental tissues and cord blood at delivery	-	Term vs. PTB: Placenta (ng/g): 0.02 (0.01) vs. 0.81 (1.43), *p*: 0.009 Maternal serum and cord blood (µg/dL): No significant association	7
Jelliffe et al., 2006 [25]	USA	Cohort	*n* = 262	Maternal blood during pregnancy	Low birth weight, race, insurance, maternal age, parity, infant sex	Pb level of <10 µg/dL vs. ≥10 µg/dL: AOR, 95% CI: 3.2, 1.2 to 7.4, *p* < 0.05	9
Li et al., 2017 [26]	China	Cohort	*n* = 3125	Maternal blood during pregnancy	Pre pregnancy BMI, maternal age, time of serum collection, gravidity, parity, and monthly income.	Medium-Pb grp (1.18–1.70 µg/dL): AOR, 95% CI: 2.33, 1.49 to 3.65 High-Pb grp (>1.71 µg/dL): AOR, 95% CI: 3.09, 2.01 to 4.76	8
Ozsoy et al., 2012 [38]	Turkey	Cross-sectional	*n* = 810	Meconium collected at birth	-	Median (min–max) (ng/g/Kg) in Term vs. PTB known etiology:10.2 (4.6–27.1) vs. 15.5 (5.8–43.2), *p* < 0.001	8
Perkins et al., 2014 [27]	USA	Cohort	*n* = 949	Maternal blood during pregnancy	Maternal age, pre-pregnancy BMI, income, maternal serum zinc concentration, gravidity and parity	Mean 1.2 µg/dL (range, 0.0–5.0) Highest vs. lowest quartile: OR, 95% CI: 1.85, 0.79 to 4.34	9
Rabito et al., 2014 [39]	USA	Cohort	*n* = 98	Maternal blood during second and third trimester of pregnancy	-	Geometric mean (range) (µg/dL): 2nd trimester: 0.43 (0.19–1.22) 3rd trimester: 0.43 (0.19–2.10) OR, 95% CI for 0.1 unit increase 2nd trimester: 1.66, 1.23 to 2.23, *p* < 0.01 3rd trimester: 1.24, 1.01 to 1.52, *p* < 0.05	8
Taylor et al., 2015 [28]	UK	Cohort	*n* = 4285	Maternal blood during pregnancy	Maternal height, smoking, parity, infant sex and gestational age.	Mean: 3.67 ± 1.47 µg/dL. <5 vs. ≥5 µg/dL: AOR, 95% CI 2.00, 1.35 to 3.00	8
Tsuji et al., 2018 [29]	Japan	Cohort	*n* = 14,847	Maternal blood during pregnancy	Pre-pregnancy BMI, smoking, partner smoking, drinking habits, gravidity, parity, the number of cesarean sections, uterine infection, household income, educational levels, and sex of infant	No significant association	8
Vigeh et al., 2011 [30]	Iran	Cohort	*n* = 348	Maternal blood during pregnancy	Maternal age, infant sex, education, passive smoking, pregnancy weight gain, parity, hematocrit and type of delivery	PTB vs. Term, means ± SD: 4.46 ± 1.86 vs. 3.43 ± 1.22 mg/dL, *p* < 0.05 OR, 95% CI:1.41, 1.08 to 1.84	8
Wai et al., 2017 [31]	Myanmar	Cohort	*n* = 419	Maternal urine during pregnancy.	Maternal age, education, infant sex, smoking, gestational age, primigravida and antenatal visits	No significant association	7
Yildirim et al., 2019 [40]	Turkey	Case-control	*n* = 50	Maternal blood and urine, amniotic fluid and cord blood	-	Term vs. PTB: Pb concentration (μg/L) Mother urine: 2.62 (1.07–3.35) vs. 1.83 (1.08–3.14), *p* < 0.001 Maternal blood, cord blood, amnion fluid: No significant association	7
Zhang et al., 2015 [32]	China	Case-control	*n* = 408	Maternal urine	Gestational age, income, maternal BMI, parity, passive smoking, and hypertension during pregnancy.	Highest tertile (≥11.67 µg/g) vs. lowest tertile (<5.41μg/g): AOR, 95% CI: 2.96, 1.49–5.87	6
Zhu et al., 2010 [33]	USA	Retrospective cohort	*n* = 43,288	Maternal blood before delivery	Maternal age, gestational age, parity, race, ethnicity, education, smoking, alcohol drinking, drug abuse, in wedlock, participation in special financial assistant program, timing of lead test, and infant sex	Mean: 2.1 µg/dL No significant association between quartiles	5

**Table 2:** Association between Mercury and PTB.

Reference	Country	Study Design	Sample Size	Specimen Type and Timing	Control Variables Adjusted	Association with PTB Odds Ratio or Relative Risk (95% CI), *p* Value	Qualitative Assessment Score
Chen et al., 2014 [41]	USA	Cohort	*n* = 50	Maternal blood and cord blood at delivery	-	Term vs. PTB: Mean (95% CI) Mother’s plasma (µg/L): 0.55 (0.48–0.63) vs. 0.93 (0.78–1.10), *p* = 0.0002 Mother RBC (µg/L): 1.37 (1.21–1.55) vs. 1.86 (1.49–2.33), *p* = 0.026 Cord plasma (µg/L): 0.46 (0.40–0.53) vs. 0.83 (0.73–0.94), *p* = 0.0024 Cord RBC (µg/L): 1.65 (1.46–1.86) vs. 2.22 (1.67–2.96), *p* = 0.039	5
Bashore et al., 2014 [42]	USA	Cohort	*n* = 159	Urine at pregnancy, cord blood	Maternal age and race	No significant association	7
Burch et al., 2014 [43]	USA	Ecological	*n* = 362,625	Hg level in fish	Mother’s age, education, race, smoking, previous live births and stillborn	OR, 95% CI For African American: 2nd quartile (>0.17–0.29 ppm): 1.14, 1.08 to 1.21 3rd quartile (>0.29–0.62 ppm): 1.18, 1.11 to 1.25 4th quartile (>0.62 ppm): 1.10, 1.04 to 1.17 For European American: 2nd quartile (>0.17–0.29 ppm): 1.06, 1.02 to 1.11 3rd quartile and 4th quartile: No significant association	-
Freire et al., 2019 [24]	Spain	Cohort	*n* = 327	Placental tissue	Maternal education, infant sex, level of other metals (As, Pb, Cd, Mn, Cr),	No significant association	8
Irwinda et al., 2019 [37]	Indonesia	Cross-sectional	*n* = 51	Maternal blood, placental tissues and cord blood at delivery	-	Term vs. PTB: Placental Hg level (ng/g): 0.20 (0.17) vs. 20.47 (41.35), *p* = 0.019 Serum and Cord blood Hg levels (µg/L): No significant association	7
Tsuji et al., 2018 [29]	Japan	Cohort	*n* = 14,847	Maternal blood during pregnancy	Pre-pregnancy BMI, smoking, partner, drinking, gravidity, parity, the number of cesarean sections, uterine infection, income, educational levels, and sex of infant	No significant association	8
Yildirim et al., 2019 [40]	Turkey	Case-control	*n* = 50	Maternal blood and urine, amniotic fluid, cord blood	-	No significant association	7

**Table 3:** Association between Cadmium and PTB.

Reference	Country	Study Design	Sample Size	Specimen Type and Timing	Control Variables Adjusted	Association with PTB Odds Ratio/Relative Risk (95% CI), *p* Value	Qualitative Assessment Score
Freire et al., 2019 [24]	Spain	Cohort	*n* = 327	Placental tissue	Maternal education, infant sex, level of other metals (Pb, As, Mn, Cr),	Median (25th and 75th percentiles) (ng/g): 4.452 (2.786–6.487) OR, 95% CI for each 10% increase of Cd: 0.92, 0.84 to 0.99	8
Huang et al., 2017 [45]	China	Case-control	*n* = 408	Urine during pregnancy	Maternal education, household income, pre-pregnancy BMI, parity and passive smoking during pregnancy	Median (range) (μg/g) Cases: 0.60, (<0.01–5.61) Controls: 0.48 (0.04–18.09) Preterm low birth weight: OR, 95% CI Medium (0.35–0.70): 1.75, 0.88 to 3.47 High (≥0.70): 2.51, 1.24 to 5.07	6
Johnston et al., 2014 [46]	USA	Cohort	*n* = 1027	Maternal blood during pregnancy	Maternal age, education, race, insurance, parity, history of anxiety, cotinine defined smoking status, and infant sex	Mean ± SD (mg/L): 0.46 ± 0.34 OR, 95% CI for one SD increase of Cd Medium (0.29–0.49 µg/L):1.24, 0.81 to 1.89 High (≥0.50 µg/L): 1.17, 0.74 to 1.87	6
Ozsoy et al., 2012 [38]	Turkey	Cross-sectional	*n* = 810	Meconium	-	Median (min-max) (ng/g/Kg) in Term vs. PTB Known etiology: 0.78 (0.28–2.57) vs. 1.31 (0.48–5.03), *p ≤* 0.001	8
Tsuji et al., 2018 [29]	Japan	Cohort	*n* = 14,847	Maternal blood during pregnancy	Pre-pregnancy BMI, smoking, partner smoking, drinking, gravidity, parity, the number of cesarean sections, uterine infection, household income, education, and infant sex	Median (ng/g) (25th and 75th percentiles) Early preterm = 0.79 (0.57,1.18) Late preterm = 0.71 (0.51,0.98) Term = 0.66 (0.50, 0.90), *p* = 0.014	8
Wai et al., 2017 [31]	Myanmar	Cohort	*n* = 419	Maternal urine during pregnancy	Maternal age, education, infant sex, smoking status, gestational age, primigravida and antenatal visits	Median (IQR): 0.86 (0.50–1.40) µg/g creatinine AOR, 95% CI for one unit increase of Cd: 1.05, 0.97 to1.13	7
Wang et al., 2016 [47]	China	Cohort	*n* = 3254	Maternal blood during pregnancy	pre-pregnancy BMI, maternal age, income, parity, gravidity and serum zinc level	Mean (range) (mg/L): 0.89 (0.04–8.08) Medium (0.65 to 0.94 mg/L) serum Cd: No significant association High serum Cd level (≥0.95 mg/L) AOR, 95% CI: 3.02, 2.02 to 4.50; *p* < 0.001	8
Yang et al., 2016 [48]	China	Cohort	*n* = 5364	Maternal urine before delivery	Maternal age, education, pre-pregnancy BMI, parity, passive smoking, net weight gain during pregnancy, infant sex, other metals (arsenic, lead)	Geometric mean (range) μg/g creatinine: 0.55 (0.01–2.85) AOR, 95% CI for each ln-unit increase in urinary Cd: 1.78, 1.45 to 2.19	9
Yildirim et al., 2019 [40]	Turkey	Case-control	*n* = 50	Maternal blood, urine, amniotic fluid and cord blood	-	No significant association	7

**Table 4:** Association between Arsenic and PTB.

Reference	Country	Study Design	Sample Size	Specimen Type and Timing	Control Variables Adjusted	Association with PTB Odds Ratio/Relative Risk (95% CI), *p* Value	Qualitative Assessment Score
Ahmad et al. 2001 [49]	Bangladesh	Cross-Sectional	*n* = 192	Tube well water	Maternal age, education, age at marriage, SES	Mean As levels: High exposure group: 0.240 mg/L Low exposure group: ≤0.02 mg/L High exposure vs. low exposure: 122.2 vs. 47.8, *p* value 0.018	7
Almberg et al. 2017 [50]	USA	Ecological	*n* = 428,804	Drinking water	Maternal age, education, marital status, parity, race/ethnicity, smoking, pre-pregnancy BMI, infant sex, Women, Infant, and Children (WIC) supplemental nutrition program,	AOR, 95% CI for1 μg/L increase in As in drinking water for counties with: ^a^ Well restriction <10:1.10, 1.06 to 1.15 ^b^ Well restriction <20: 1.08, 1.02 to 1.14	-
Banu et al. 2013 [51]	Bangladesh	Ecological	*n* = 321	Tube well water	Maternal age, education, weight gain during pregnancy, environmental tobacco smoke, pregnancy history, and spouse’s education	No significant association	-
Freire et al. 2019 [24]	Spain	Cohort	*n* = 327	Placental tissue	Maternal education, infant sex, cohort (random effect), all other metals.	No significant association	8
Myers et al. 2010 [52]	China	Cross-Sectional	*n* = 9890	Well water	Adequacy of prenatal care utilization	No significant association	7
Rahman et al. 2018 [53]	Bangladesh	Cohort	*n* = 1183	Tube well water and toenail samples	Maternal age, education, enrollment BMI, number of past pregnancies, passive smoking, and water arsenic exposure.	Median (range) As levels for drinking water (µg/L): 2·2 (<LOD–1400) Toenail samples (µg/g): 1.2 (<LOD–46.6) RR, 95% CI for one unit increase in natural log As For drinking water: 1.12, 1.07 to 1.17 For toenail: 1.13, 1.03 to 1.24	9
Shi et al. 2015 [54]	USA	Ecological	*n* = 177,995	Ground water	-	PTB when arsenic level >10 µg/L: r = 0.70	-
Wai et al. 2017 [31]	Myanmar	Cohort	*n* = 419	Maternal urine during pregnancy	Maternal age, education, infant sex, smoking, gestational age, primigravida and antenatal visits	Median (IQR): 74 (45–127) µg/g creatinine AOR, 95% CI for one unit increase of As: 1.00, 0.99 to1.00	7
Wang et al. 2018 [55]	China	Cohort	*n* = 3194	Maternal blood	Pre-pregnancy BMI	Mean, median (range) (µg/L): 5.10, 4.87 (0.02 to 43.52) High As group (>6.68 μg/L) OR, 95% CI: 1.47, 1.03 to 2.09, *p* = 0.034	6
Yang et al. 2003 [56]	Taiwan	Ecological	*n* = 18,259	Well water	Maternal age, education, marital status, and infant sex	No significant association	-

^a^ 8−10% increase in the odds of PTB per 1 μg/L increase in arsenic in drinking water for counties with <10% private well use. ^b^ 8−10% increase in the odds of PTB per 1 μg/L increase in arsenic in drinking water for counties with <20% private well use.

## Data Availability

Not applicable.
